# TMT-based proteomics analysis of the blood enriching mechanism of the total Tannins of Gei Herba in mice

**DOI:** 10.1016/j.heliyon.2024.e33212

**Published:** 2024-06-17

**Authors:** Wenbi Mu, Cancan Duan, Jingwen Ao, Fanpan Du, Jianyong Zhang

**Affiliations:** a Zunyi Product Quality Inspection and Testing Institute, Zunyi, 563000, China; b Department of Pharmaceutical Analysis, School of Pharmacy, Zunyi Medical University, Zunyi, 563000, China; c Pharmacology, Key Laboratory of Basic Pharmacology of Ministry of Education and Joint International Research Laboratory of Ethnomedicine of Ministry of Education, Zunyi Medical University, Zunyi, 563000, China

**Keywords:** Proteomics, Total tannins of gei herba, Anemia

## Abstract

Lanbuzheng (LBZ) is the traditional seedling medicine in Guizhou, which has the effect of tonifying blood. It has been found that the main active ingredient is tannin, however, the blood-replenishing effect of tannin and its mechanism are still unclear.

The study was to explore the mechanisms underlying the therapeutic effects of the total Tannins of Lanbuzheng (LBZT) against anemia in mice. Anemia mice was induced by cyclophosphamide, the effect of LBZT against anemia was determined by analyzing peripheral blood and evaluating organs indexes. Tandem mass tag (TMT)-based quantitative proteomics technology coupled with bioinformatics analysis was then used to identify differentially expressed proteins (DEPs) in spleen. Compared to the model, number of RBCs, PLTs and WBCs, HCT ratio and HGB content were increased, the indexes of thymus, spleen and liver were also increased, after LBZT intervention. A total of 377 DEPs were identified in LBZT group, of which 206 DEPs were significantly up-regulated and 171 DEPs were significantly down-regulated. Bioinformatics analysis showed that hematopoietic function has been restored by activating the complement and coagulation cascade signaling pathways. Results suggest that LBZT exerts it therapeutic effects against anemia by regulating complement and coagulation cascade signaling pathways and provides scientific basis for further mechanistic studies for LBZT.

## Highlights

• LBZT effectively improved anemia in cyclophosphamide (CYC)-induced mice.
• Mechanisms of protection involve upregulating of peripheral blood and organs indexes.
• Activation of complement and coagulation cascade signaling pathways underlies the protection afforded by LBZT.

## 1 Introduction

Cancer is associated with extremely high morbidity and mortality and is the second highest threat to human health [1]. Chemoimmunotherapy is a long-term maintenance therapy that plays an important role in cancer treatment. However, chemoimmunotherapy can induce certain side effects that affect the quality of life of the patients. Anemia is the main side effect of high-dose chemotherapy, which may cause thyroid diseases, and acute myeloid leukemia [2,3]. Currently, the primary methods used to treat chemotherapy-induced anemia include blood transfusion as well as iron, and erythropoietin supplementations [4]. However, recent clinical research revealed that the use of recombinant human Granulocyte-macrophage Colony Stimulating Factor (rh-GM-CSF) and iron chelating agents were associated with individual differences, easy tolerance and certain side effects, limiting their use [5]. Therefore, there is an urgent need to find new drugs for the treatment of chemotherapy-induced anemia.

Traditional Chinese medicines (TCM) have been widely used as blood tonics for thousands of years due to their unique advantages such as having multiple targets and low side effects [6,7]. For instance, Colla corii asini and Danggui Buxue Decoction have been widely used as blood tonics in the clinical setting [[8], [9], [10]]. However, the complexity of TCM components limits their clinical application. Therefore, there is a great potential in the use of TCM to develop therapeutic agents for anemia.

Gei Herba (Chinese name: Lanbuzheng, LBZ) is the dried whole grass of *Geum japonicum* Thunb. var. *Chinense* Bolle and *Geum aleppicum* Jacq, which is included in the Chinese Pharmacopoeia (2020 edition). In our previous studies, we demonstrated that the aqueous extracts from Gei Herba were able to restore dysfunctional hematopoiesis in anemic mice by regulating JAK2/STAT3 signaling pathway in spleen [11,12]. These results indicated that aqueous extracts from Gei Herba had pharmacodynamic properties. Further analysis revealed that tannins, polysaccharide and volatile oils were the major components of Gei Herba [13]. Among these components, tannins have been reported to improve immunity and treat several ischemic diseases [14]. However, there are few studies on the effects of the total Tannins of Gei Herba (LBZT) on anemia, and the related mechanisms of action remains unclear.

Quantitative proteomics test is a precise technique that can be used to identify differentially expressed proteomics (DEPs) involved in the development of diseases. The technique can also be used to identify drug targets and their mechanisms of action [15,16]. Tandem mass tag-based (TMT) is a quantitative proteomic technique that can label and analyze multiple biological samples with high sensitivity and generate high-quality data [17,18]. In addition, TMT is commonly used to study the therapeutic targets and mechanisms of action of TCMs [19,20].

In this study, TMT quantitative proteomics technique was employed to explore the targets and the related signaling pathways of LBZT in anemic mice. Bioinformatics analysis was then used to identify the important targets and the mechanisms of actions associated with LBZT-induced therapeutic effect against anemia.

## 2 Material and methods

### 2.1 LBZT preparation

LBZ was collected from Zunyi City, Guizhou Province, China. Which was identified as *Geum japonicum* Thunb. var. *Chinense* Bolle by Dr Nie Xuqiang (Department of Pharmacy, Zunyi Medical University, China), and stored in the college of Pharmacy.

LBZ powder (100 g) was soaked in 50 % acetone (AR, lot: 20190801, Chongqing Chuandong Chemical Co., ltd) solution overnight at a ratio of 1:10 (g:mL) at room temperature. Ultrasonic extraction for 10 min, filtration, and concentration (120 rpm, 45 °C) with rotary evaporator (lot: STRIKE 280 M4, Jolabo Technology (Beijing) Co., ltd) until no odor. Then, water was added to 500 mL and extracted with ethyl acetate (AR, lot number:20190504, Chengdu Jinjin Chemical Test Co., ltd.), the upper liquid was concentrated, added water to 300 mL and filtered. 90 mL gelatin solution (0.33 g/mL) was added to obtain precipitation, added 250 mL 90 % acetone solution, ultrasonic extraction for 2 h, the solution was concentrated (120 rpm, 45 °C), and LBZT powder was obtained through vacuum drying. The lyophilized powder was dissolved in distilled water to a solution of 2 mg mL^−1^ (w/v), and then stored at 4 °C.

### 2.2 Materials and reagents

Acetone (AR, lot: 20190801, Chongqing Chuandong Chemical Co., ltd), ethyl acetate (AR, lot: 20190504, Chengdu Jinshan Chemical Test Co., ltd), RIPA lysate (lot：R0010，Solarbio, Beijing), SDS-PAGE (lot: S8010, Solarbio, Beijing), PVDF (lot: R7EA8786A, Millipore Trading, USA), mouse anti-β-actin (lot: 60008-1-lg, Proteintech, Chicago, USA), mouse anti-C8 (lot: ab231255, Abcam, Cambridge, UK), anti-mouse IgG (lot: SA00001-2, Proteintech, Chicago, USA), rotary evaporator (lot: STRIKE 280 M4, Jolabo Technology (Beijing) Co., ltd), HPLC (Agilent Technologies1260 Series, 5 μm, 4 × 250 mm, USA).

### 2.3 Quality control of LBZT

LBZT (30 mg) was soaked overnight in an erlenmeyer flask containing 40 mL of 50 % acetone and ultrasound for 10 min, and all filtrates were concentrated at 45 °C, 120 rpm by a rotary evaporator (lot: STRIKE 280 M4, Jolabo Technology (Beijing) Co., ltd) to obtain 10 ml of concentrated liquid. Then add water to make dilute 10 times the concentration of the liquid to be tested, mixed and reserved. The amount of total polyphenols, free polyphenols and casein blank solution polyphenols were determined according to a protocol we had previously published [21]. Finally, the content of LBZT (c-LBZT) was calculated.

$$c-\text{LBZT}=\frac{\text{LBZT}}{\text{LBZ}}\times 100\%$$

LBZT was accurately weighed (equivalent to the relative mass of 1 g crude drug) and then dissolved in 75 % methanol in a 100 mL corked conical flask. Afterwards, the solution was filtered and then analyzed using high performance liquid chromatography HPLC (Agilent Technologies1260 Series，USA) chromatograph coupled with UV-VIS detector and an HPLC column Agilent Eclipese XDB-C18 (5 μm, 4 × 250 mm). An external standard method was used to determine the amounts of ellagic acid and gallic acid in LBZT [22].

### 2.4 Animals and drug administration

Specific pathogen-free (SPF) Kunming (KM) female mice (3–4 weeks, 18–22 g) were supplied by the SiPeiFu (Beijing) Biotechnology Co.,Ltd. The animals received food and water at liberty, were housed three per cage and were acclimatized to the environment under standard conditions (12-hr day-night cycle, room temperature 23 ± 2 °C, room relative humidity 40%–60 %) for more than 7 days. All animal experimental protocols were approved by the Institutional Committee on Animal Care and Use of Zunyi Medical University.

The mice were randomly divided into 3 groups: control group, anemia model group and LBZT group (8 per group). The anemia mice model was induced based on our previous research [12]. Briefly, anemia was induced in the mice in the model and LBZT groups through intraperitoneal injection of cyclophosphamide (CYC) on the 8th to 10th of gavage. Thereafter, mice were administered with saline solution in control and anemia model groups, while mice in LBZT group were intragastrically administered with equal amounts of LBZT (20 mg/kg) for 13 days.

#### 2.4.1 Peripheral blood analysis and organ index detection

Blood samples (0.5 mL) were collected into centrifuge tubes containing 50 μL EDTA-2Na (15 mg/mL) 2 h after administration of the last dose of treatment. The blood samples were used to determine the peripheral blood indexes including hemoglobin (HGB), red blood cell count (RBC), white blood cell count (WBC), haematocrit (HCT) and platelet count (PLT). Thymus, spleen and liver samples were also isolated and weighed. The organ indexes (thymus, spleen and liver) were calculated based on the relationship between organ weight (OW) and body weight (BW). The organ samples were kept at −80 °C until further analysis.

$$\text{OI}=\frac{\text{OW}\left(mg\right)}{\text{BW}\left(g\right)}$$

### 2.5 TMT-based proteomics analysis

#### 2.5.1 Protein preparation and TMT labeling

Nine spleen cortices (3 spleens per group) were selected for TMT quantitative proteomics detection. Frozen spleen tissues were lysed in lysis buffer (4 % SDS, 100 mM Tris-HCl, 1 mM DDT, pH 7.6, 14000×*g*, 4 °C), centrifuged, and the supernatant collected. Protein concentration was quantified using BCA Quantification kit (Bio-Rad, USA) according to the instruction. Thereafter, 0.5 μg/μL trypsin solution was used to digest the protein extracted from each sample, and labeled with TMT using a 10-plex TMT kit. Peptides in each group were labeled with different TMT labels: three biological repeats of control group were labeled as TMT-126, TMT-127 N and TMT-127C; three biological repeats of model group were labeled as TMT-128 N, TMT-128C and TMT-129 N; three biological repeats of LBZT group were labeled as TMT-129C, TMT-130 N and TMT-130C. The labeled samples were mixed, vacuum dried, and stored at −20 °C for further analysis.

#### 2.5.2 High pH reversed-phase fractionation and LC-MS/MS analysis

TMT labeled peptides were dissolved in 100 μL buffer A (98 % double-distilled water, 2 % acetonitrile, pH 10) and fractionated via high pH reversed-phase fractionation chromatography using an EASY-nLC-1000 HPLC System (Beijing RIGOL Technologies Inc., Beijing, China). Fractions were collected in 20 tubes every 1.75 min and then dried and combined into 10 tubes for further LC-MS/MS analysis.

LC-MS/MS analysis was performed using an EASY-nLC1000 System (Nano HPLC, Thermo Fisher Scientific, USA) coupled to a Q-Exactive Mass Spectrometer with an EASY-Spray Ion Source (Thermo Fisher Scientific, USA). Samples were loaded onto an Acclaim PepMap 100 precolumn (2 cm × 100 μm, 5 μm, C18, Thermo Fisher Scientific, USA). Peptide separation was conducted using an EASY-Spray column (12 cm × 75 μm, 3 μm, C18, Thermo Fisher Scientific, USA) with buffer A (100 % ultrapure water and 0.1 % formic acid) and buffer B (100 % acetonitrile and 0.1 % formic acid) at a flow rate of 0.3 mL/min.

#### 2.5.3 Proteomics identification and quantitative analysis

The LC-MS/MS raw data were analyzed using the MASCOT engine (Matrix Science, London, UK; version 2.2) embedded into Proteome Discoverer GE Healthcare software for identification and quantification. The false discovery rate (FDR) was set as <0.01. Detection of at least one unique peptide per protein was set as the requirement for protein identification. The protein quantitative analysis was based on reporter ion peak intensity. Differentially expressed proteomics (DEPs) were identified through ratio-fold change as well as *P* value calculated with a *t*-test, and set at an average ratio-fold change >1.2 (up-regulation) or < 0.83 (down-regulation) with a *P* value < 0.05. The proteins that satisfied the set thresholds were considered to be “Differentially expressed proteomics” (DEPs) and were used for further bioinformatics analysis.

#### 2.5.4 Bioinformatics analysis

Functional annotations of DEPs were performed through Gene Ontology (GO) enrichment analysis (http://www.geneontology.org/). Proteins were classified by GO annotation based on three categories: biological process (BP), cellular component (CC) and molecular function (MF). For each category, a two-tailed Fisher's exact test was used to test the enrichment of the DEP against all identified proteins. A corrected *P*-value <0.05 was considered significant in the GO analysis. The Search Tool for the Retrieval of Interacting Genes (STRING, version 10.5) database was used to perform protein-protein interaction network analysis of DEPs. Only the experimentally validated interactions with a minimum required interaction score >0.7 was considered as interaction network.

### 2.6 Western blot for validation of complement component 8

Spleen tissues were added to 300 μL ice-cold RIPA lysate [12] (lot：R0010，Solarbio, Beijing) to prepare the tissue homogenates, lysed on ice for 15 min, and centrifuged (12000 rpm, 4 °C). The proteins were separated (80 V, 30 min) in 10 % sodium dodecyl sulfate–polyacrylamide gel electrophoresis (SDS-PAGE, lot: S8010, Solarbio, Beijing) and transferred to nitrocellulose membranes. Next, PVDF (lot: R7EA8786A, Millipore Trading, USA) membrane was sealed for 120 min (120 V), and then each mixture was incubated with individual antibody overnight at 4 °C on a shaker. The first antibody information was as follows: mouse anti-β-actin (1:5000, Proteintech, Chicago, USA), mouse anti-C8 (1:1000, Abcam, Cambridge, UK). After being washed 3 times with PBST (15 min each time), the membranes were incubated with horseradish peroxidase (HRP) conjugated goat anti-mouse IgG secondary antibody (1:5000, Proteintech, Chicago, USA) at room temperature. The proteins were finally examined using an enhanced chemiluminescence system (Bio-Rad Laboratories, Inc., Hercules, CA, USA). Image lab (version 3.0) was used to analyze the bands, and the relative expression level of each target protein was calculated using β-actin as an endogenous reference.

### 2.7 Statistics

All data were expressed as mean ± standard deviation (SD). SPSS software (version 18.0) was used for statistical analysis. Results were compared using independent *t*-test or one-way analysis of variance (ANOVA). A *P*-value <0.05 was considered statistically significant.

## 3 Results and discussion

### 3.1 Chemical analysis of LBZT

**Fig. 1:**
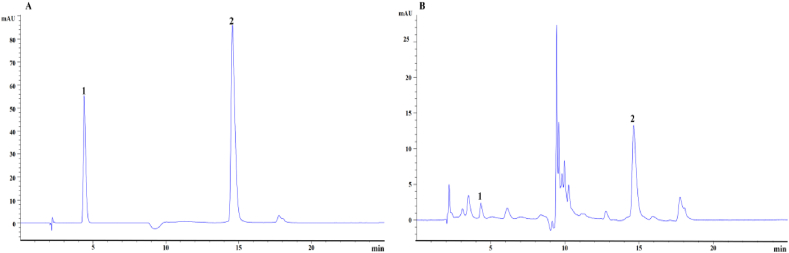
HPLC chromatogram of LBZT. Note: (A) standard solution of gallic acid and ellagic acid; (B) sample; “1, 2” represents gallic acid and ellagic acid respectively.

In this study, 6.85 g LBZT was extracted from 100 g LBZ giving a yield of 0.068 %, and a concentration of 725.71 mg/g. HPLC analysis revealed that the amount of gallic acid and ellagic acid in LBZT was 41.24 and 352.56 mg/g, respectively (Fig. 1A–B). Gallic acid and ellagic acid are the main components of LBZT.

### 3.2 Peripheral blood and organ indexes analysis

**Fig. 2:**
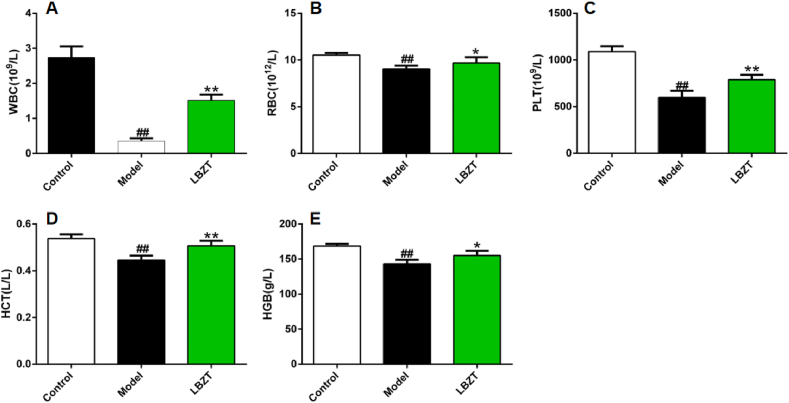
Effects of LBZT on peripheral blood indexes of hemorrhagic anemia mice ($\bar{x}\pm S,n=6$). Note: A: WBC, B: RBC, C: PLT, D: HCT, E: HGB, control vs model, ##P < 0.01; model vs LBZT, *P < 0.05, **P < 0.01.

There was a significant decrease in the levels of WBC (Fig. 2A), RBC (Fig. 2B), PLT (Fig. 2C), HCT (Fig. 2D) and HGB (Fig. 2E) in the model group compared to the control group (*P* < 0.01). In contrast, the levels of WBC (Fig. 2A), RBC (Fig. 2B), PLT (Fig. 2C), HCT (Fig. 2D) and HGB (Fig. 2E) were significantly higher in the LBZT group compared to the model group (*P* < 0.01 or *P* < 0.05).

**Fig. 3:**
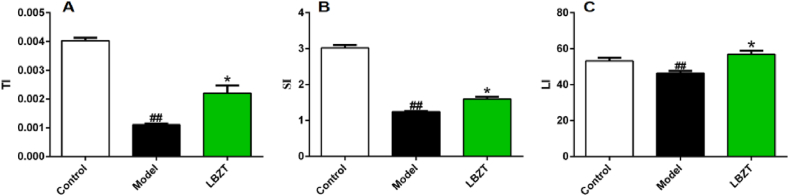
Effect of LBZT on organ index in mouse of anemia($\bar{x}\pm S,n=6$**)**. Note: A: Thymus index, B: Spleen index, C: Liver index, control vs model, ##P < 0.01; model vs LBZT, *P < 0.05.

Additionally, the organ indexes of the thymus (Fig. 3A), spleen (Fig. 3B) and liver (Fig. 3C) in the model group were significantly lower than those of the control group (*P* < 0.01). On the other hand, mice treated with LBZT had significantly higher organ indexes of thymus, spleen and liver compared to the model group (*P* < 0.05).

### 3.3 TMT analysis proteins of spleen

**Fig. 4:**
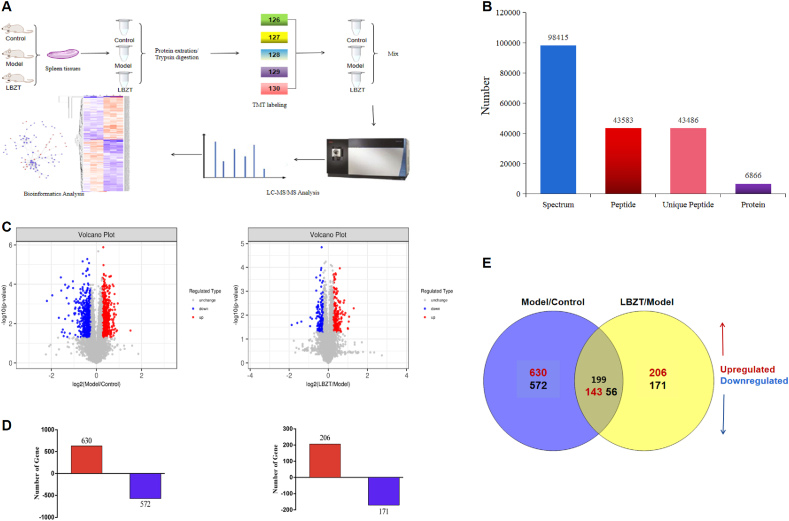
TMT-based quantification of the anemia. Note: (A) Workflow for TMT protein profiling of anemia in this study. (B) Protein identification results. 43,486 proteins (unique peptides≥1) were identified from 48,583 unique peptides. (C) Volcano of DEPs among the two comparison groups. (D) The number of up-and down-regulated proteins in each comparison group. (E) Venn diagram of the distribution and overlaps of DEPs among the two comparison groups.

**Fig. 5:**
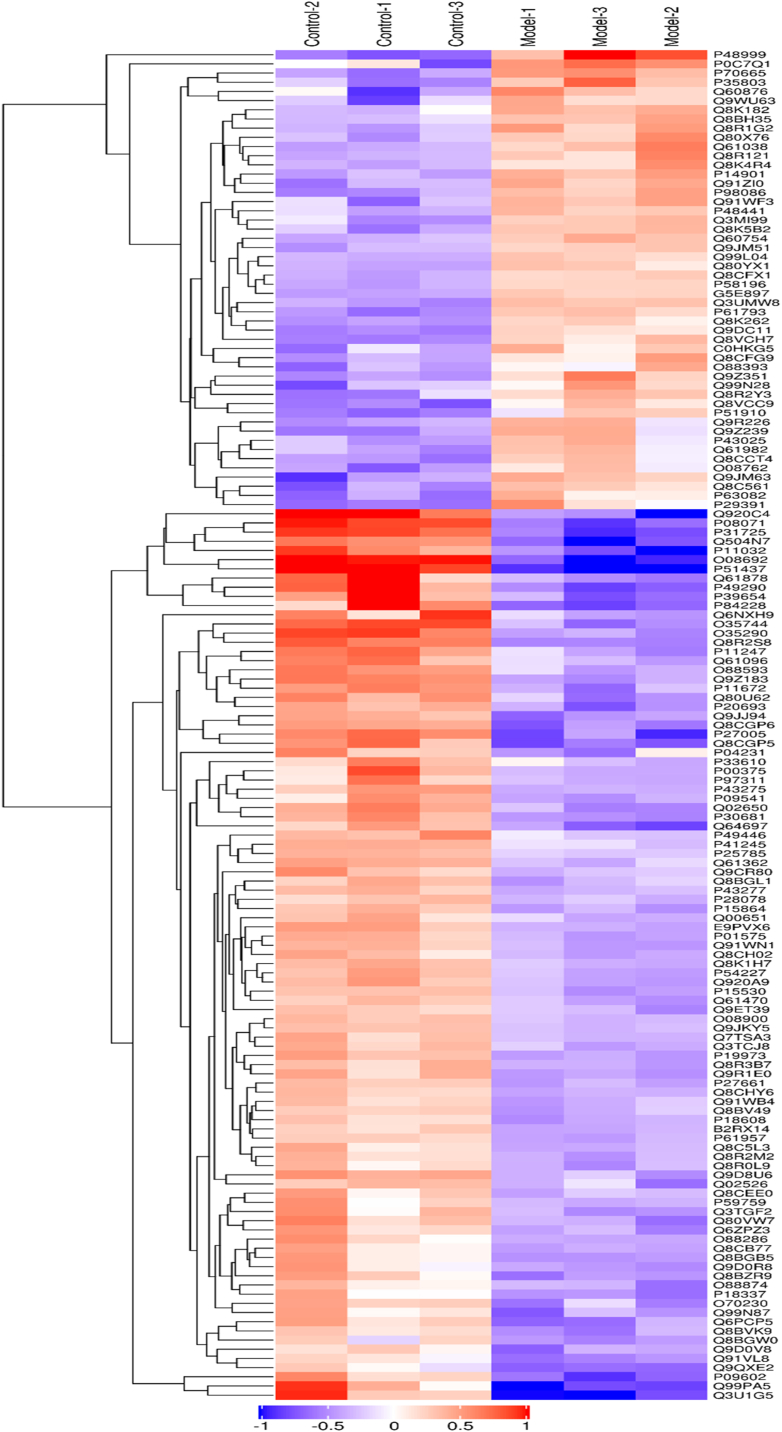
Hierarchical clustering of 199 common differentially expressed proteins**.** Note: the orange represented significantly up-regulated proteins, and the purple represented significantly down-regulated proteins. (For interpretation of the references to colour in this figure legend, the reader is referred to the Web version of this article.)

To further identify the mechanisms of LBZT-induced therapeutic effects against anemia, TMT-based quantitative proteomics assay and bioinformatics analyses were performed. Fig. 4A is the workflow of this study LC-MS/MS results shown that a total of 98,415 spectrum were obtained, 48,583 proteins (unique peptides ≥1) were identified from 43,486 unique peptides, and 6809 proteins were successfully quantified (Fig. 4B). The volcano maps of DEPs are shown in Fig. 4C. A total of 1202 DEPs (630 up-regulated and 572 down-regulated) were identified between the model group and the control group, while 377 DEPs (206 up-regulated and 171 down-regulated) were identified between the LBZT treated group and the anemia model group (Fig. 4D). The distribution and overlap of DEPs between the two comparison groups are shown in the Venn diagram (Fig. 4E). There were 199 common DEPs among these comparison groups, including 143 up-regulated proteins and 56 down-regulated proteins. The expression of these common DEPs was analyzed through hierarchical clustering (Fig. 5).

### 3.4 Bioinformatics analysis of DEPs

**Fig. 6:**
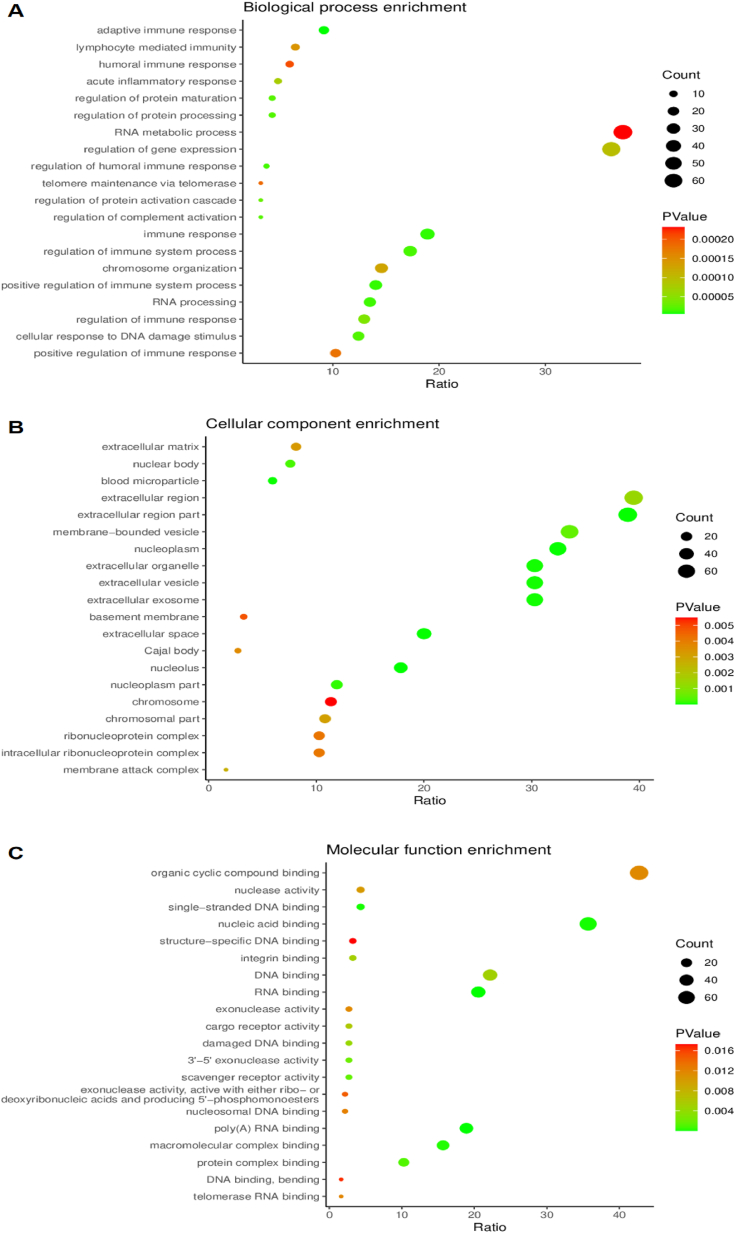
Gene ontology (GO) enrichment analysis of 199 common differentially expressed proteins. Note: (A) Top 20 biological process enrichment terms. (B) Top 20 cellular component enrichment terms. (C) Top 20 molecular function enrichment terms. The size of the spot represents the number of genes corresponding to each term, and the colour represents−log(Pvalue). (For interpretation of the references to colour in this figure legend, the reader is referred to the Web version of this article.)

To clarify the biological significance of the 199 DEPs, GO enrichment analysis was performed to annotate the functions of these proteins according to CC, MF and BP. The DEPs were significantly enriched in 628 GO terms (*P* < 0.05), with 38 terms being in the cellular component, 32 in molecular function, and 232 in biological process. The top 20 terms of each category are shown in Fig. 5. Regarding BP, the DEPs were enriched in the regulation of the adaptive immune system, which might play important roles in regulating immunity (Fig. 6A). From the CC terms, the DEPs were mainly located in nucleolus (Fig. 6B). In MF, poly(A) RNA binding was the top enriched term, while receptor binding contained the most DEPs (Fig. 6C).

### 3.5 KEGG pathway and PPI analysis of DEPs

Further analysis of the biological functions of the DEPs was carried out using KEGG pathway enrichment analysis. Table 1 showed that 6 KEGG pathways including systemic lupus erythematosus, complement and coagulation cascades, staphylococcus aureus infection, prion diseases, base excision repair and pertussis were obtained.

**Table 1:** KEGG Pathway enrichment analysis of 199 DEPs.

Term	Count	PValue	Genes
Systemic lupus erythematosus	10	2.27E-06	C1QA, C5, FCGR2A, HIST1H2AH, H3F3C, H2AFX, C8B, C8A, HIST2H2AC, C1QC
Complement and coagulation cascades	7	2.94E-05	C1QA, C5, CFH, SERPING1, C8B, C8A, C1QC
Staphylococcus aureus infection	6	1.05E-04	C1QA, C5, FCGR2A, CFH, ICAM1, C1QC
Prion diseases	5	2.13E-04	C1QA, C5, C8B, C8A, C1QC
Pertussis	4	0.028528476	C1QA, C5, SERPINE1, C1QC
Base excision repair	3	0.034247233	XRCC1, POLL, HMGB1

**Fig. 7:**
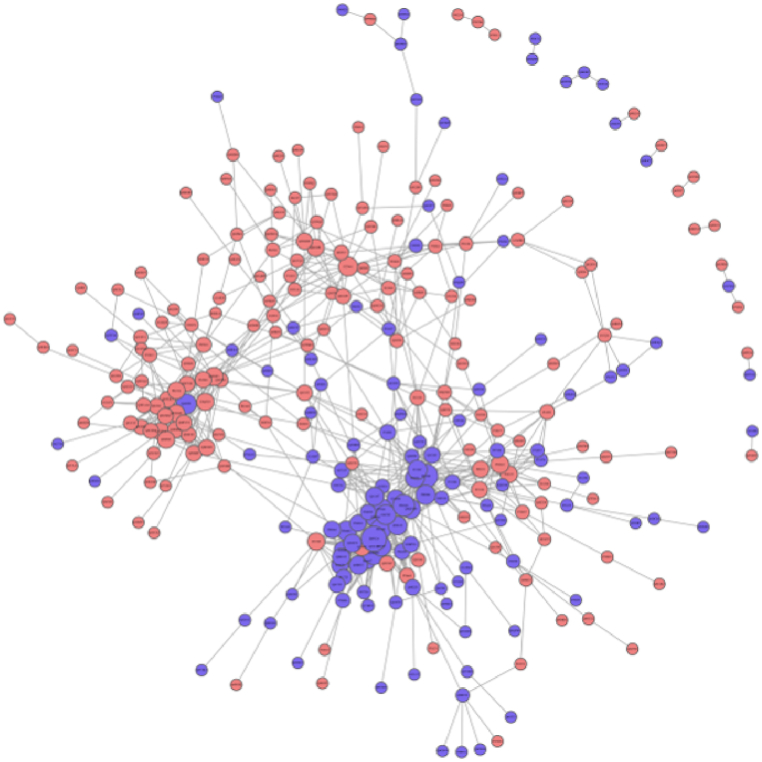
PPI network analysis. Note: the orange circle represented significantly up-regulated proteins, and the blue represented significantly down-regulated proteins. (For interpretation of the references to colour in this figure legend, the reader is referred to the Web version of this article.)

Additionally, network analysis identified complex interactions (either direct or indirect) between these proteins. The result of PPI analysis showed that orange circle represented significantly up-regulated proteins, and the blue represented significantly down-regulated proteins (Fig. 7).

**Fig. 8:**
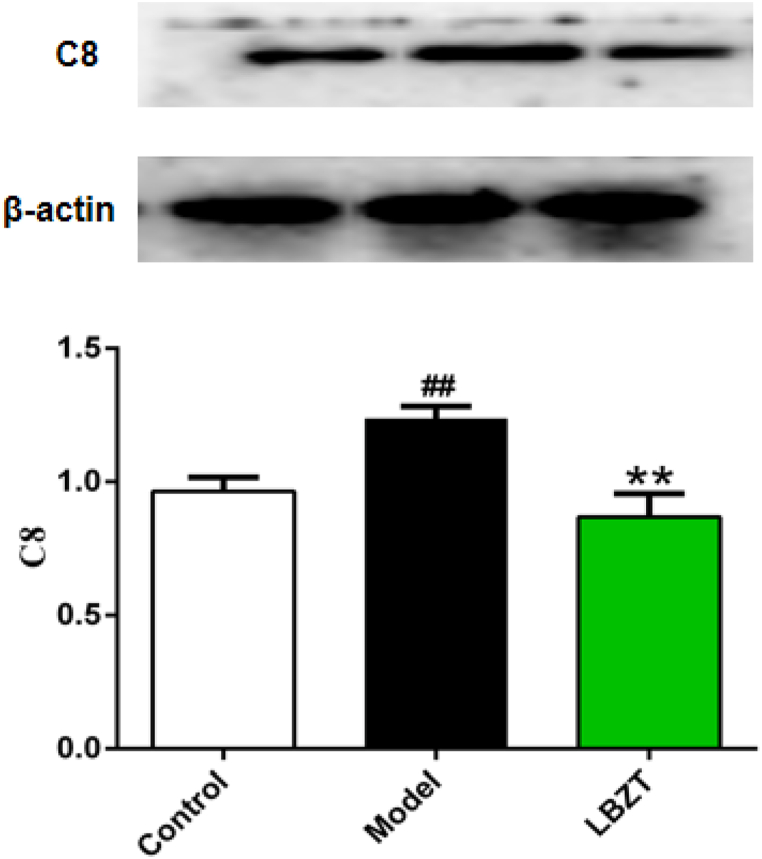
Validation of C8. Note: Model vs Control, ##P < 0.01; model vs LBZT, **P < 0.01.

Of these DEPs, C8 was selected to verify the reliability of quantitative proteomics. C8 is one of the most important targets in complement and coagulation cascades pathways. The results showed that the expression of C8 was up-regulated in the anemia model group compared with the control group, but was significantly down-regulated in the LBZT group (Fig. 8) compared with the model group. These findings were consistent with the results of proteomics data.

## 4 Discussion

In this study, a CYC-induced anemia mice model was established to explore the protective effects and blood enrichment mechanisms of LBZT using a proteomic and biochemical analysis approach.

In our previous study, we demonstrated that aqueous extracts of Gei Herba restored the hematopoietic function of mice with CYC-induced blood deficiency. We further showed that the extracts played a role in supplementing blood by regulating peripheral blood, organ index and hematopotietic factors in spleen as well as activating the JAK2/STAT3 signaling pathway [12]. Additionally, the results of metabolomics and network pharmacology have shown that LBZ improves blood deficiency in mice [11,12]. However, the components responsible for the blood tonic effects of LBZ have not been identified. Therefore, in this study, we further extracted LBZT from the LBZ water extracts and explored the mechanisms of its blood enrichment effects. Our results showed that LBZT administration increased the levels of WBC, HCT, PLT, RBC, and HGB as well as the thymus, spleen and liver indexes to alleviate the symptoms of anemia. These results revealed that LBZT has blood enrichment properties and exerts therapeutic effects against anemia by improving the peripheral blood indexes and organ indexes.

Modern pharmacology has shown that the spleen is an extramedullary hematopoietic organ that can restore hematopoietic function under certain pathological conditions [23]. Studies have found that anemia is closely linked to the spleen, and that the spleen index is an effective indicator for evaluating blood tonic effects of drugs [24]. For example, hemorrhagic anemia in rats was significantly improved by regulating splenic proteins and metabolites using Danggui Buxue Decoction [20]. To further reveal the mechanisms of LBZT-induced protective effects against the spleen in ameliorating anemia, a TMT-based quantitative proteomics analysis was performed to globally screen LBZT protein targets. The results obtained from this analysis were validated using bioinformatics analysis. Our results indicated that LBZT exerts therapeutic effects against anemia by regulating the expression of complement C1QA, C1QC, C5, C8A and C8B and the subsequent complement and coagulation cascades.

Complement is an important immune regulatory factor with multiple functions, such as regulating inflammation, processing self-antigens and regulating the expression of immune-related genes. Complement also participates in biological processes such as apoptosis and autophagy for maintaining body homeostasis [25]. Several studies have shown that complement plays a core role in maintaining homeostasis and defending against pathogens in the tissue [26,27]. Additionally, complement is a focal immunomodulatory target for treating inflammatory diseases [28]. Studies found that non-severe aplastic anemia could be cured by increasing the level of complement C1Q in the serum of the patients [25]. C5 is one of the important complement components in the inflammatory response. C5a is a cleavage product of C5 that mediates the inflammatory response, while C5b also participates in the regulation of the immune response [29]. The membrane attack complex (MAC) plays an important role in resisting exogenous microorganisms and clearing the immune defense response of infected host cells. C8 is a component of MAC that directly destroys the integrity of the target cell membrane and can cause immune diseases [30]. In this study, we observed a significant increase in the levels of C1QA, C1QC, C5, C8A and C8B in the spleen of anemic mice, which was an indication that the immune system of the anemic mice had been compromised. Administration of LBZT led to a significant reduction in the levels of the complement molecules, which suggested that LBZT induced protective effects in anemic mice by inhibiting the over expression of complement components.

The complement system mediates inflammation, immune regulation, and the clearance of immune complexes [31]. The coagulation and complement system were ancestrally related to enzymatic cascades of the blood, and the complement system is a proteolytic cascade in blood and mediator of innate immunity [32]. Further, the complement system has emerged as an attractive target for early and upstream intervention in inflammatory diseases [33]. In addition, there is evidence that anemia decreases immune function and induces inflammatory response [34]. Based on the above findings, we speculated that the complement system may affect hematopoietic function by regulating immunity and inflammation.

More meaningful, a study found that anemia could cause abnormalities in hematopoietic processes and immune responses [35]. KEGG pathway enrichment results from our study showed that LBZT induced protective effects against the spleen of anemic mice by regulating the complement and coagulation cascade signaling pathways. These findings indicated that LBZT can boost immunity and facilitate recovery of hematopoiesis in individuals with anemia.

## 5 Conclusion

In this study, our results revealed that LBZT exerts therapeutic effects against anemia by regulating 199 DEPs, particularly those involved in the complement and coagulation cascade pathway. This work provides insights into further proteomic studies on the therapeutic effects of LBZT against anemia.

## 6 Ethics statement

All procedures including animal use were approved by the official Review Board at Princess Nourah University, Riyadh, KSA (IRB Number 20–0391), which are follow those established by the US National Institutes of Health (NIH publication No. 85–23, revised 1996).

## Funding

This research was funded by the 10.13039/501100001809National Natural Science Foundation of China (Grants no. 82360819,82360768,81560736), Guizhou science and Technology Department program of China (QKH platform talents [2020] 5007,YQK[2023]038,ZK[2022]-588) and Science and Technology Department of Zunyi city of Guizhou province of China ([2021]-3).

## Data availability statement

The [DATA TYPE] data used to support the findings of this study are included within the article.

## CRediT authorship contribution statement

**Wenbi Mu:** Validation, Writing – original draft, Writing – review & editing. **Cancan Duan:** Software, Supervision. **Jingwen Ao:** Conceptualization, Investigation, Methodology. **Fanpan Du:** Investigation, Methodology. **Jianyong Zhang:** Project administration, Resources.

## Declaration of competing interest

The authors declare that they have no known competing financial interests or personal relationships that could have appeared to influence the work reported in this paper.
