# Supplementary material for: TMT-based proteomics analysis of the blood enriching mechanism of the total Tannins of Gei Herba in mice
Source: Heliyon. 2024 Jun 17;10(12):e33212. doi: 10.1016/j.heliyon.2024.e33212 (PMC11253055; doi:10.1016/j.heliyon.2024.e33212)
Supplement: Multimedia component 1 [file mmc1.docx]

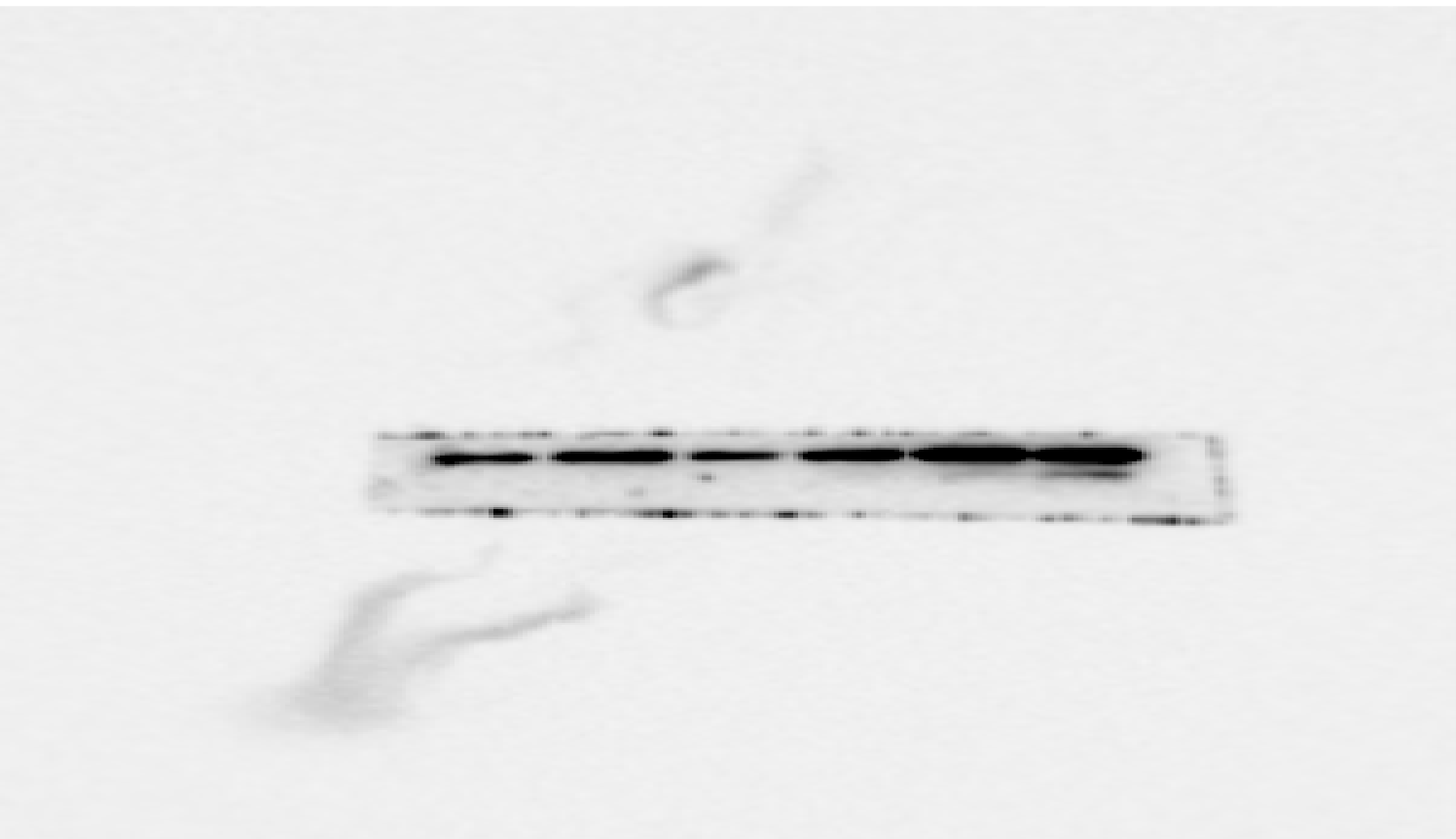


**Shown in text**

**LBZT**

**Model**

**Control**

**LBZT**

**Model**

**Control**

**Validation of C8**

**
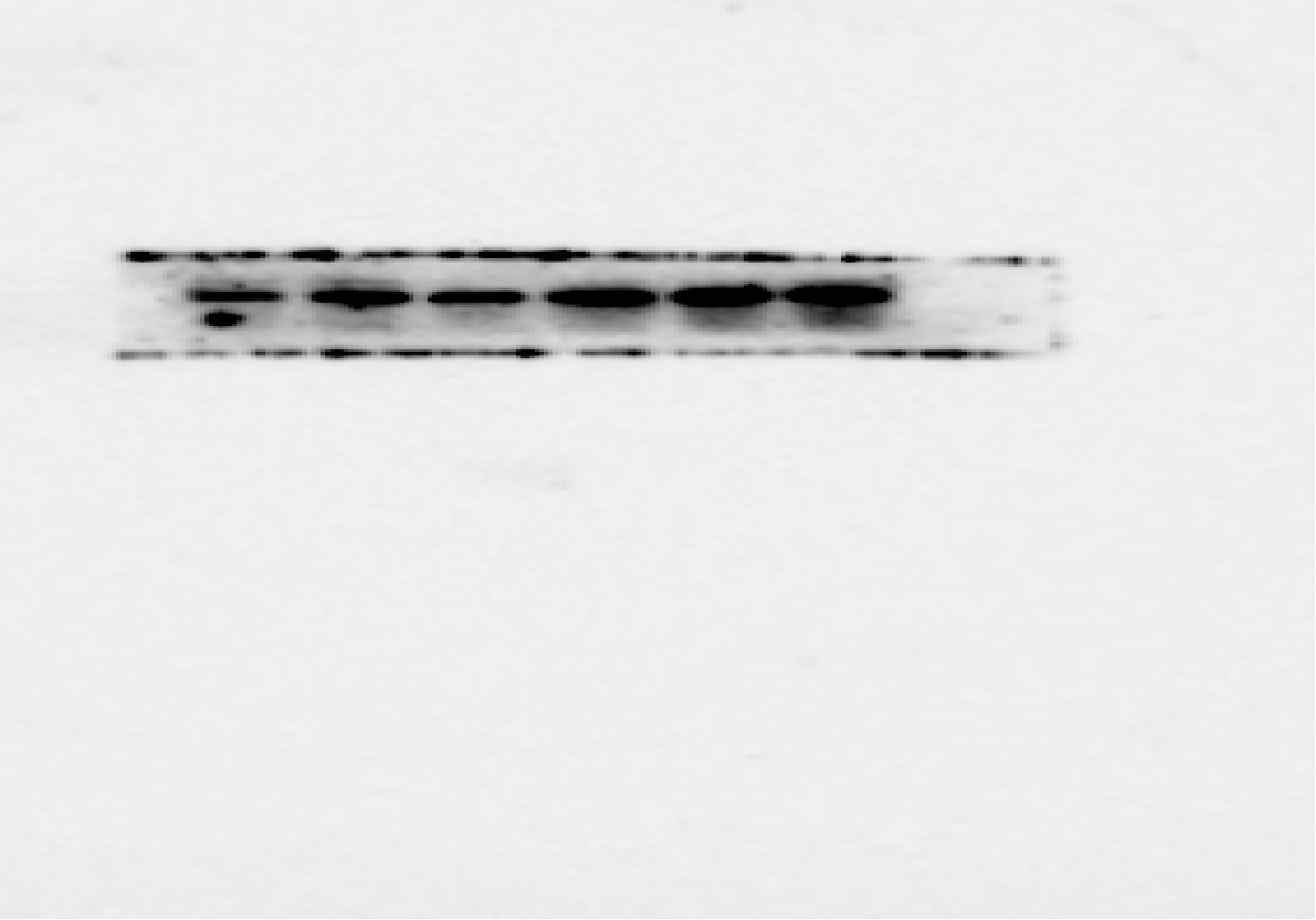
**

**Shown in text**

**LBZT**

**Model**

**Control**

**Model**

**LBZT**

**Control**

**Validation of β-actin**

The uncropped versions of figure [8] in text.
